# Cloning, Expression, and Characterization of a Thermophilic Endoglucanase, AcCel12B from *Acidothermus cellulolyticus* 11B

**DOI:** 10.3390/ijms161025080

**Published:** 2015-10-22

**Authors:** Junling Wang, Gui Gao, Yuwei Li, Liangzhen Yang, Yanli Liang, Hanyong Jin, Weiwei Han, Yan Feng, Zuoming Zhang

**Affiliations:** 1 Key Laboratory for Molecular Enzymology & Engineering of the Ministry of Education, School of Life Science, Jilin University, Changchun 130012, China; E-Mails: wangjl09@mails.jlu.edu.cn (J.W.); gaogui@jlu.edu.cn (G.G.); liyw11@mails.jlu.edu.cn (Y.L.); yanglz13@mails.jlu.edu.cn (L.Y.); liangyl13@mails.jlu.edu.cn (Y.L.); hyjin13@mails.jlu.edu.cn (H.J.); weiweihan@jlu.edu.cn (W.H.); yfeng2009@mail.sjtu.edu.cn (Y.F.); 2 Department of Biotechnology, Jilin Agricultural Science and Technology College, Jilin 132101, China; 3 State Key Laboratory of Supramolecular Structure and Materials, College of Chemistry, Jilin University, Changchun 130012, China

**Keywords:** *Acidothermus cellulolyticus 11B*, endoglucanase, AcCel12B, thermostability, reaction slowdown

## Abstract

The gene *ABK52392* from the thermophilic bacterium *Acidothermus cellulolyticus 11B* was predicted to be endoglucanase and classified into glycoside hydrolase family 12. *ABK52392* encodes a protein containing a catalytic domain and a carbohydrate binding module. *ABK52392* was cloned and functionally expressed in *Escherichia coli*. After purification by Ni-NTA agarose affinity chromatography and Q-Sepharose^®^ Fast Flow chromatography, the properties of the recombinant protein (AcCel12B) were characterized. AcCel12B exhibited optimal activity at pH 4.5 and 75 °C. The half-lives of AcCel12B at 60 and 70 °C were about 90 and 2 h, respectively, under acidic conditions. The specific hydrolytic activities of AcCel12B at 70 °C and pH 4.5 for sodium carboxymethylcellulose (CMC) and regenerated amorphous cellulose (RAC) were 118.3 and 104.0 U·mg^−1^, respectively. The *K*_m_ and *V*_max_ of AcCel12B for CMC were 25.47 mg·mL^−1^ and 131.75 U·mg^−1^, respectively. The time course of hydrolysis for RAC was investigated by measuring reducing ends in the soluble and insoluble phases. The total hydrolysis rate rapidly decreased after the early stage of incubation and the generation of insoluble reducing ends decreased earlier than that of soluble reducing ends. High thermostability of the cellulase indicates its potential commercial significance and it could be exploited for industrial application in the future.

## 1. Introduction

Cellulose, one of the most important components of plant cell walls in the biosphere, is composed of a linear backbone composed of glucose units linked by β-1,4-d-glucosidic bonds. Cellulolytic microorganisms degrade crystalline cellulose by producing a comprehensive cellulase system consisting of three classes of enzymes: endoglucanases (EGs, EC 3.2.1.4), cellobiohydrolases (CBHs, EC 3.2.1.91), and β-glucosidases (BGLs, EC 3.2.1.21) [1]. In addition, polysaccharide monooxygenases are newly found cellulose-degrading enzymes mainly distributed in cellulolytic fungi and actinomycete bacteria [2]. The biodegradation of cellulose requires the synergistic action of these cellulolytic enzymes, which have been used in industrial fields including biofuels, brewing, textiles, and laundry detergents, and to improve the processing of paper pulp, the de-inking of paper, and the efficiency of using plant materials in animal feeds [3,4]. For industrial applications, thermophilic and hyperthermophilic enzymes generally present some advantages over mesophilic ones, for example, higher thermal, pH, and chemical stabilities, higher reaction temperatures, and higher catalytic rates. These properties are useful for the enzymatic hydrolysis of lignocellulosic materials [5].

EGs randomly hydrolyze accessible intramolecular bonds of cellulose chains to generate soluble oligosaccharides and new free chain ends, while CBHs processively release soluble cellobiose or glucose from the free ends [6]. Thus, EGs play an important role in the synergistic hydrolysis of cellulose together with CBHs. Glycoside hydrolase family 12 (GH12) enzymes hydrolyze glycosidic bonds by a double displacement mechanism, in which the configuration of the anomeric carbon (C1) is retained after hydrolysis [7]. A number of GH12 enzymes from thermophilic bacteria (including *Thermotoga neapolitana* and *Rhodothermus marinus*) and hyperthermophilic archaea (including *Sulfolobus solfataricus* and *Pyrococcus furiosus*) were characterized. The enzymes presented high thermal stability and resistance to low pH. The temperature optima for CelA and CelB from *T. neapolitana* were 95 and 110 °C, respectively [8]. The enzymes from *P. furiosus* and *R. marinus*, which both showed a temperature optimum of 100 °C, were also extremely thermostable [9,10]. All of the above enzymes showed optimal activity at neutral pH. In contrast, SSO1949 from *S. solfataricus* showed a half-life of about 8 h at 80 °C and pH 1.8, which could be valuable properties for the large-scale hydrolysis of cellulose under high temperatures and acidic conditions [11].

*Acidothermus cellulolyticus* was first isolated from acidic hot springs in Yellowstone National Park, USA. The bacteria efficiently used cellulose as a carbon source, were resistant to acid environments (pH 4–6), and showed optimal growth between 37 and 70 °C [12]. A few cellulolytic enzymes from *A. cellulolyticus* have previously been characterized. For example, xylanase from *A. cellulolyticus* (Xyn10A) presented optimal activity at 90 °C and pH 6 [13]. In particular, the endoglucanase E1 (GH5) has received much attention due to its demonstrated high thermostability, with activity at temperatures up to 81 °C, and its high specific activity for CMC [14]. Endoglucanase E1 has been successfully expressed in various cells, for instance tobacco and *Caldicellulosiruptor bescii* [15,16]. *A. cellulolyticus* also has other endoglucanase genes, for instance ABK52392 and ABK52388. These two genes were predicted to encode endoglucanase with multiple domains that have been classified into GH12 (http://www.cazy.org) [17,18]. The catalytic domain of ABK52392 was successfully expressed in *Zymomonas mobilis* but the properties of the recombinant protein have not been reported so far [19]. In this work, the endoglucanase encoded by the gene ABK52392 from *A. cellulolyticus* was expressed in *E. coli* and the recombinant protein (AcCel12B) was purified and characterized. The results suggested that AcCel12B was thermostable in acidic conditions, and the time course of hydrolysis for insoluble substrates was investigated.

## 2. Results and Discussion

### 2.1. Architecture Analysis and Sequence Alignment

**Figure 1:**
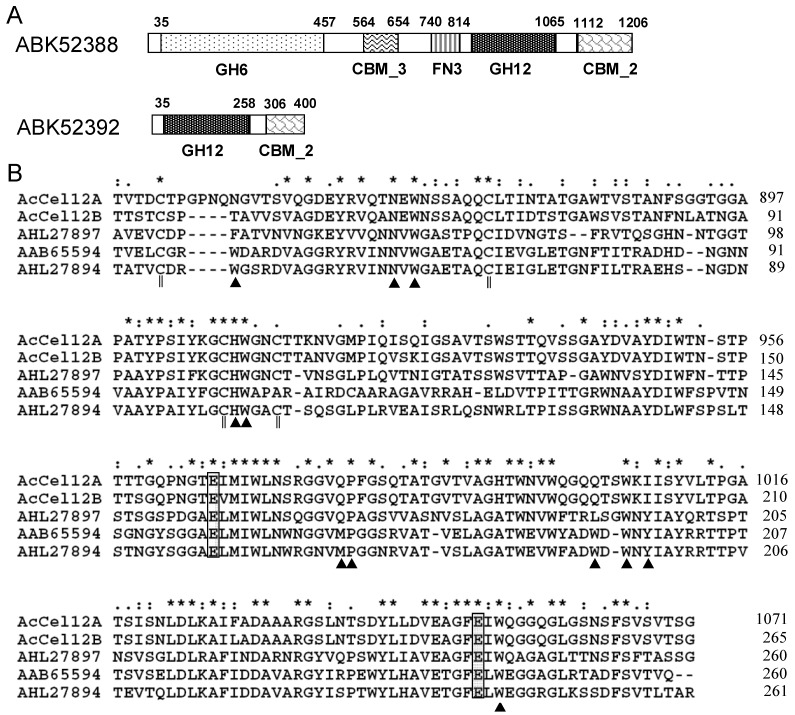
Domain schematics of ABK52388 and ABK52392 (**A**) and multiple alignment of catalytic domain sequences (**B**). (**A**) Putative signal peptides (N-terminal open boxes), glycoside hydrolase family domains (GH6 or GH12), carbohydrate binding modules (CBM_3 or CBM_2) and the FN3 domain are shown using different patterned boxes and the positions of the modules are shown by numbers above the sequences; (**B**) Catalytic sites (gray boxes), substrate binding sites (black triangles), and four cysteines that formed two disulfide bonds (double vertical lines), identical residues in all five sequences (*), highly conserved residues (:), weakly conserved column (.). AcCel12A and AcCel12B, the CD of GH12 from *A. cellulolyticus*; AHL27897 and AHL27894, the CD of GH12 from environmental samples; AAB65594, the CD of GH12 from *R. marinus* ITI378.

The genome of *A. cellulolyticus* was sequenced [12] and ABK52392 and ABK52388 were predicted to encode endoglucanases [17,18]. Each of the proteins encoded by the two genes consisted of multiple domains. The domain architecture was performed using the SMART tool (http://smart.embl-heidelberg.de/) [20]. As indicated in Figure 1A, ABK52392 was comprised of a catalytic domain (CD) with homology to GH12 and a carbohydrate binding domain (CBM_2) connected by a long and proline/serine-rich linker. The domain architecture of ABK52392 was quite similar to that of the C-terminal half of ABK52388. The protein sequences of ABK52392 (residues 36–403) and the C-terminal half of ABK52388 (residues 838–1209) were compared using the BLAST program [21]. The two proteins shared sequence identity as high as 85%. Accordingly, the sequence of ABK52392 was compared with other characterized cellulases of GH12. The result indicated that ABK52392 has a 48% identity to the cellulase AHL27897 isolated from the GH12 metagenome but very low identity to other GH12 proteins. However, the CD sequence of ABK52392 showed a slightly higher identity to other GH12 members; 54% to AHL27897, 43% to AHL27894, and 39% to AAB65594, for instance. The CD sequences were aligned using the Clustal X software program (Figure 1B). The two catalytic sites of AHL27897 (Glu158 and Glu241, AHL27894 numbering), which were confirmed by determining the structure of the protein in complex with the substrate [22], were strictly conserved. Some of the substrate binding sites (Asn58, Trp60, His101, Trp102, Pro171, Trp195, and Trp243) of AHL27897 were conserved in ABK52388 and ABK52392 but the other sites (Trp43, Met170, Trp193, and Tyr197) were not conserved.

### 2.2. Cloning, Expression, and Purification of AcCel12B

**Figure 2:**
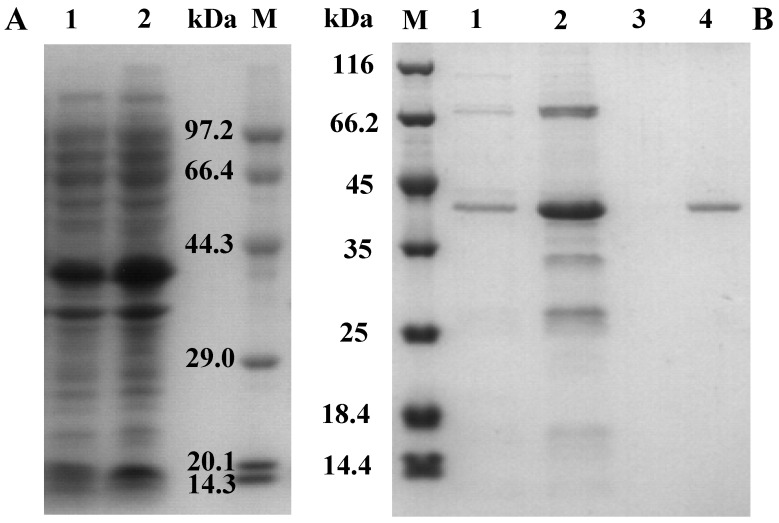
SDS-PAGE gel electrophoresis to confirm the expression (**A**) and purification (**B**) of recombinant protein AcCel12B. (**A**) 1, Supernatant of the cell lysate; 2, Pellet of the cell lysate; M, Protein marker; (**B**) M, Protein marker; 1–3, elutions in 50, 80, and 150 mM imidazole, respectively; 4, AcCel12B purified by Q-Sepharose^®^ Fast Flow chromatography.

The gene encoding AcCel12B (without signal peptides) was amplified by PCR using genomic DNA of *A. cellulolyticus* 11B as template and inserted into plasmid pET-20b (+). After verifying the sequence, the reconstructed plasmid was transformed into *Escherichia coli* BL21-CodonPlus (DE3)-RIL. Expression of the recombinant protein, AcCel12B, was induced by treatment with 0.5 mM isopropyl β-d-1-thiogalactopyranoside (IPTG) at 16 °C. As indicated in Figure 2A, both the soluble and insoluble forms of the recombinant protein were overproduced. The molecular weight of the recombinant protein was congruent with the predicted molecular weight of the mature protein (38.3 kDa). AcCel12B (C-terminal fused His-tag) was purified by Ni-NTA agarose affinity chromatography and Q-Sepharose^®^ Fast Flow (Uppsala, Sweden) chromatography, and the yield and purification fold were 1.1% and 98, respectively. The homogeneity of the purified protein was verified by sodium dodecyl sulfate polyacrylamide gel electrophoresis (SDS-PAGE) (Figure 2B).

### 2.3. Enzyme Properties of AcCel12B

#### 2.3.1. Effects of pH and Temperature on AcCel12B Activity

**Figure 3:**
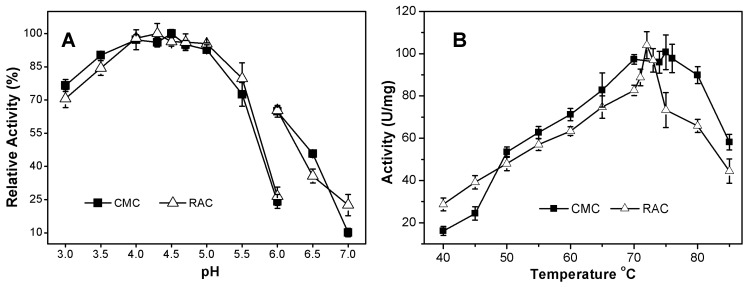
Effects of pH (**A**) and temperature (**B**) on the activity of AcCel12B. (**A**) 50 mM sodium acetate buffer (pH 3.0–6.0), 50 mM sodium phosphate buffer (pH 6.0–7.0); (**B**) Activity was determined in 50 mM sodium acetate buffer (pH 4.5). AcCel12B was incubated with 1% (*w*/*v*) CMC or 0.5% (*w*/*v*) RAC for 5 or 15 min, respectively. The formation of reducing sugars was then determined. The relative activity was calculated as a percentage of the maximal activity. All data were averaged from triplicate measurements.

The pH optimum of the purified recombinant AcCel12B was determined by incubating the enzyme in buffers of different pH containing CMC or RAC as substrate. As indicated in Figure 3A, AcCel12B exhibited activity over a broad range of pH (3.0–7.0) with optima at pH 4.5 for CMC and pH 4.3 for RAC. It retained more than 80% activity over the pH range of 3.5–5.0, demonstrating its high activity under acidic conditions. Some archaeal and fungal cellulases in GH12 also presented high activity under acidic conditions. SSO1949 and EG II, for instance, showed their highest activities at pH 1.8 and pH 3.5, respectively [11,23]. In contrast, bacterial cellulases in GH12 have commonly been reported to show their highest activities under near-neutral pH conditions. The pH optima of endocellulase CelB and Cel12A from *T. neapolitana* and *R. marinus* were pH 6.0–6.6 and pH 7.5, respectively [8]. On the other hand, the enzyme Cel12A from the alkaliphilic *Streptomyces* sp. 11AG8Ac showed carboxymethylcellulase activities over a broad range of pH (5–10) with an optimum activity at pH 8 [24]. The temperature dependence of activity was assayed by incubating Cel12B with CMC (1%, *w*/*v*) or RAC (0.5%, *w*/*v*) for 5 or 15 min at various temperatures (Figure 3B). At temperatures ranging from 40 to 70 °C, the relative activity of AcCel12B increased with each incremental rise in temperature; however, a rapid reduction in activity was observed when temperature was raised beyond 73 °C and RAC was used as substrate. The temperature optima for AcCel12B activity were determined to be 75 °C for CMC and 72 °C for RAC. It retained more than 50% relative activity for both substrates at temperatures between 50 and 85 °C. Reductions in the activity of AcCel12B towards RAC were observed at lower and higher temperatures (70 and 75 °C, for instance) compared with its activity towards CMC. One reason for this finding may arise from partial degradation of the CBM, and another reason may arise from the different incubation times used when assaying the activity of AcCel12B towards RAC and CMC. Therefore, the recombinant AcCel12B was active under acidic pH and high temperature conditions. These characteristics correspond with the fact that *A. cellulolyticus* is a thermophilic and acidophilic bacteria whose optimal growth pH and temperature were shown to be pH 5 and 55 °C, respectively [12].

#### 2.3.2. Thermal Stability of AcCel12B

**Figure 4:**
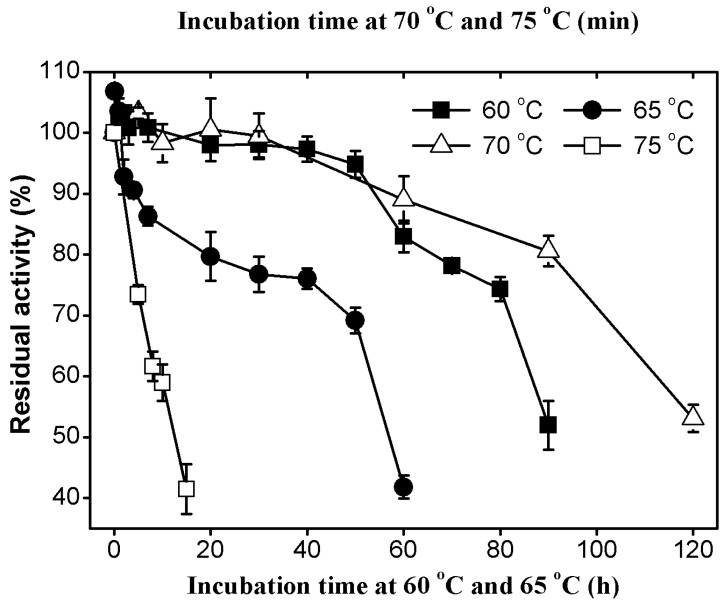
Thermal stability of AcCel12B at 60, 65, 70, and 75 °C. AcCel12B was incubated at 60 °C (**■**), 65 °C (**●**), 70 °C (∆) and 75 °C (**□**) in 50 mM sodium acetate buffer (pH 4.5) for various lengths of time. Residual activity was assayed using CMC as substrate. All data were averaged from triplicate measurements.

AcCel12B was incubated at various temperatures for prolonged durations at pH 4.5 and the residual activity were then assayed (Figure 4). The enzyme was extremely stable at 60 °C and two days of incubation at this temperature did not lead to a significant decrease in cellulase activity. The half-lives of AcCel12B at 60, 65, and 70 °C were about 90, 55, and 2 h, respectively. The enzyme was less stable at 75 °C, at which temperature the half-life dropped to 12 min. The cellulases from thermophiles were commonly showed high thermal stabilities. RmCel12A from the thermophilic bacteria *R. marinus* was reported to have a half-life of 2.5 h at 90 °C [25]. In contrast, EglA from the hyperthermophilic archaeon *P. furiosus* was reported to have a half-life as long as 40 h at 95°C [10]. Moreover, the enzyme was deactivated quickly and its activity was reduced by 76% after 30 min at neutral pH. The thermal unfolding of AcCel12B at pH 4.5 and pH 7.0 was determined by differential scanning calorimetry (Figure S1). The result indicated that the *T*m values of AcCel12B at pH 4.5 and pH 7.0 were 76.5 and 63.5 °C, respectively. This observation was quite similar to the previously reported data for SSO1949 and EG II [11,23]. SSO1949 showed a thermal inactivation rate of nearly 100 times higher at pH 7 than at pH 2.

#### 2.3.3. Effects of Metal Ions and Chemical Agents on AcCel12B Activity

The effects of metal ions and chemical agents on AcCel12B activity were determined using CMC as substrate (Table S1). As indicated in Table S1, AcCel12B presented 112% and 108% relative activity when incubated with 1 and 10 mM Co^2+^, respectively, while Cd^2+^ slightly reduced the enzyme activity. The other metal ions had no obvious effects on enzyme activity. Ethylenediaminetetraacetic acid (1 or 10 mM) and Tween^®^ 20 (1%, *v*/*v*) reduced the enzyme activity by about 10%. The GH12 endocellulase (EGPf) from the archaeon *P. furiosus* was found to contain an atypical tetragonal bipyramid geometry Ca^2+^-binding motif (DxDxDG motif) [26]. Ca^2+^-binding dramatically increased the thermostability of EGPf but had no effect on its activity. Various divalent and monovalent metal ions also had no effect on the activity of AcCel12B, suggesting that the introduction of a similar Ca^2+^-binding motif into AcCel12B might increase its thermostability.

### 2.4. Substrate Specificity and Catalytic Kinetics

The substrate specificity was determined by incubating the enzyme with various substrates at 70 °C and pH 4.5. Recombinant AcCel12B had the highest specific activity toward barley β-glucans (β-1,3-1,4-linkages) (Table 1). This property was quite similar to those of other GH12 endoglucanases [27,28,29,30]. AcCel12B also presented high activity toward CMC, RAC, and Avicel^®^ (Fluka, Buchs, Switzerland), but had no activity toward laminarin (β-1,3 linkage), starch (α-1,4 linkage), or xylan (β-1,4 linked xylose subunits). These results indicated that AcCel12B specifically hydrolyzed β-1,4 linked β-glucans, which is a trait similar to those of several other family 12 EGs [27,31,32]. AcCel12B had high activity toward insoluble β-glucans and its specific activity toward RAC was three-fold higher than its activity toward Avicel^®^, indicating that AcCel12B likely attacked the amorphous regions of insoluble β-glucans. The ability of the enzyme to hydrolyze a crystal substrate (Avicel^®^) might arise from the enzyme’s CBM on its C-terminal. Endoglucanases lacking CBM have commonly been reported to have either no or trace activity toward insoluble crystal substrates [10,23,33,34]. When the CBM was fused to the catalytic module of one of these enzymes, the chimeric cellulase showed high activity toward crystalline cellulose [35,36]. AcCel12B showed the same level of specific activity toward the soluble and insoluble substrates CMC and RAC in this study (Table 1). In contrast, other GH12 endoglucanases have commonly been found to present quite different activities toward soluble and insoluble substrates. The activity of EglA toward CMC was over 14-fold and 145-fold higher than its activities toward Whatman^®^ paper and Avicel^®^, respectively [10]. Two GH12 endoglucanases (CelA and CelB) from *T. neapolitana* presented 130-fold and 40-fold higher activity, respectively, toward CMC than toward amorphous cellulose [8]. Cel12A purified from *Gloeophyllum trabeum* showed activity toward CMC over 800-fold higher than that toward amorphous insoluble cellulose (phosphoric acid swollen cellulose) [37].

**Table 1:** Substrate specificity of AcCel12B.

Substrate	Specific Activity (U·mg^−1^)	Relativity Activity (%)
CMC (low viscosity)	118.3 ± 5.18	100
CMC (medium viscosity)	100.7 ± 8.19	85
β-d-Glucan (barley)	271.2 ± 5.79	229
RAC	104.0 ± 6.40	88
Avicel^®^ PH101	14.7 ± 0.52	12
Sigmacell cellulose Type 50	Trace	-
Pachyman	ND	-
Starch	ND	-
Xylan (birch)	ND	-
Galactomannan	ND	-

One unit of activity is defined as 1 µmol of glucose equivalents (for glucan substrates) or xylose equivalents (for xylan substrates) released per min. All assays were performed at 70 °C and pH 4.5. ND, not detected; -, not determined.

The kinetic parameters of AcCel12B were determined using CMC as substrate (supplementary data, Figure S2). The *K*_m_ and *V*_max_ were calculated to be 25.47 mg·mL^−1^ and 131.75 U·mg^−1^, respectively. AcCel12B had similar apparent *K*_m_ values to those of the enzymes CelA and CelB, but had about 10-fold lower *V*_max_ values [8]. The specific activity of AcCel12B toward insoluble amorphous cellulose was over 10-fold and 2-fold higher than those of CelA and CelB, respectively. Considering that CelA and CelB both lack a CBM, these findings suggested that the CBM of AcCel12B might play an important role in its hydrolytic activity toward amorphous cellulose.

### 2.5. Time Course of Hydrolysis

**Figure 5:**
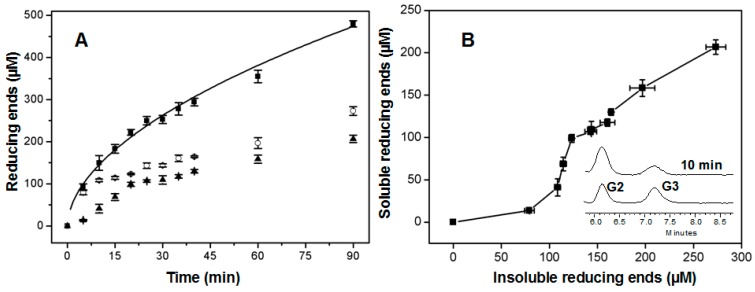
Time course of the hydrolysis of RAC by AcCel12B (**A**) and the distribution of the soluble and insoluble forms of reducing ends (**B**). (**A**) AcCel12B was incubated with RAC and the soluble (▲) and insoluble (○) reducing ends formed at the indicated time points were measured. The total amount of reducing ends generated (■) was then calculated and fitted to Equation (1) (solid line). RAC incubated with thermally inactivated AcCel12B was used as a blank. All data were averaged from triplicate measurements; (**B**) The relationship between the concentrations of soluble and insoluble ends during the reaction is shown. The insert shows representative HPLC chromatograms for the analysis of soluble products after 10 min incubation.

Enzyme catalysis was examined by incubating AcCel12B with RAC for various durations of time and then measuring the amounts of reducing sugars in the supernatant and pellet (Figure 5A). The hydrolytic generation of reducing sugars from substrate by cellulases was previously reported to be best fitted by a power function as described by Kostylev *et al.* [38].

(1) $$P_{\mathrm{tot}}=At^{b}$$

where *P*_tot_ is the total generated product (reducing ends in this case), *A* is the activity of the enzyme, *t* is the time, and *b* is the hydrolysis power factor. As indicated in Figure 5A, the power function fitted quite well (*R*^2^ = 0.991) to the generation of reducing ends over time. The parameters *A* and *b* in the equation (1) were calculated to be 42.1 ± 3.1 and 0.54 ± 0.02 μM/min, respectively. In theory, the endoglucanase should undertake multiple reaction steps to hydrolyze insoluble cellulose, theoretically including random cleavage of the amorphous region of cellulose to generate insoluble reducing ends (IRE) and soluble reducing ends (SRE), and hydrolysis of the generated soluble oligosaccharides to produce SRE. The above equation fitted the generation rate of total reducing ends, while it did not fit the individual generation rates of soluble and insoluble reducing ends by AcCel12B. The concentrations of IRE and SRE rapidly increased during the early stage of incubation and then almost linearly increased during the later stage. IRE rose to 109 μM within the first 10 min of incubation and then almost linearly increased to 273 μM during the next 80 min of incubation. The rate of SRE formation was also higher during the first 20 min than it was during the later phase of incubation. Thus, the hydrolysis of insoluble substrates by AcCel12B could be roughly divided into an initial rapid phase and a later slow phase. Under our analytical conditions, the rate of reducing ends generation during the initial phase was about five-fold (IRE) and three-fold (SRE) higher than that during the slow phase. The rate of enzymatic cellulose hydrolysis has commonly been reported to rapidly decrease after initial digestion [39]. Bansal *et al.* [40,41] reviewed this topic and suggested that no generalization could be made regarding the origin of the slowdown, concluding that substrate heterogeneity and enzyme inactivation (including nonproductive enzyme binding and noncellulosic components adsorption) were the main causes of decreasing hydrolysis rates. Murphy *et al.* [42] suggested that the slowdown was linked to the transient inactivation of endoglucanase on the cellulose surface. Shu *et al.* [43] recently revealed that the slowdown of endoglucanase (Cel5A) activity was not correlated with adsorption but was anticorrelated with the initial activity of the enzyme. An interesting finding in our study was that the generation rates of IRE and SRE at the early stage of the reaction were quite different (Figure 5B). The formation rate of IRE during the first 10 min was about two-fold higher than that of SRE. Furthermore, the slowdown of IRE formation occurred sooner than that of SRE formation; IRE formation dropped off at 10 min while SRE formation dropped off at 20 min. In order to explain why the formation rate of IRE dropped off sooner than that of SER, the apparent processivity (the ratio of the total concentration of reducing ends to that of IRE) [44] was then calculated at each indicated incubation time. It was found that the processivity was not constant within the first 20 min of incubation; the processivity increased from 1.38 at 10 min to 1.80 at 20 min. After 20 min, the processivity remained around 1.80. Some processive endoglucanases were found in GH9 and GH5. Their processivities were commonly found to be more than 3.5 [45,46]. Thus, the hydrolysis of RAC by AcCel12B did not occur via procession at either the initial rapid phase or the later slow phase. This result indicated that the observed declines in the rate of IRE formation did not arise from processive hydrolysis. A possible reason might be that the more easily accessible amorphous region of RAC was rapidly hydrolyzed by AcCel12B during the initial phase [47]. Furthermore, SRE might have been generated from IRE and/or the longer soluble oligosaccharides. The main soluble products generated during the initial phase (10 min) were analyzed by HPLC (Figure 5B insert). The result indicated that the main products were cellobiose and cellotriose, no soluble oligosaccharides with longer chain lengths were observed; thus, we concluded that SRE were solely generated from IRE. The formation of IRE was important for SRE generation. During the initial phase, AcCel12B rapidly formed readily accessible ends for SRE formation. When the IRE formation rate declined, the SRE formation rate then decreased because the concentration of attractive ends was depleted and as a result the overall hydrolysis rate was then slowed down.

## 3. Experimental Section

### 3.1. Recombinant DNA Techniques

*A. cellulolyticus* 11B was grown at 55 °C as described by Barabote *et al.* [12] and the genome was isolated using a Genomic DNA Purification Kit (AxyPrep, Shanghai, China). The gene (*ABK52392*) predicted to encode an endoglucanase was amplified by PCR with PrimeSTAR^®^ HS DNA Polymerase (TaKaRa Bio, Dalian, China) and primers (sense 5′-GCAATTCATATGTCAACGTGTTCACCTACCG-3′ and antisense 5′-TGAGACCTCGAGGCAGGTGAGTGTGGGTGGGGTGTTA-3′; *Nde*I and *Xho*I sites underlined). A PCR program was used as described by Chen *et al.* [48]. The PCR product was digested separately with *Nde*I and *Xho*I restriction enzymes (New England Biolabs, Beverley, MA, USA) and then ligated into the vector pET-20b (Novagen, Madison, WI, USA) using T_4_ DNA ligase (New England Biolabs). The recombinant plasmid was then transformed into *E. coli* BL21-CodonPlus (DE3) (Novagen) in Luria broth (LB) containing 50 μg·mL^−1^ ampicillin.

### 3.2. Expression and Purification of AcCel12B

For AcCel12B expression, cells were grown to an OD_600_ of 0.5–0.6 in LB medium containing 100 μg·mL^−1^ ampicillin at 37 °C. Protein expression was induced by incubating the cells with 0.5 mM IPTG overnight at 16 °C. The cells were harvested and resuspended in buffer A (50 mM sodium phosphate, pH 8.0). The cells were disrupted by ultrasonic treatment and the cell debris was removed by centrifugation. The supernatant of the crude extract was loaded onto a Ni-NTA column (Qiagen, Valencia, CA, USA) that had been equilibrated with buffer A and eluted using imidazole at 50, 80, or 150 mM concentrations. The protein pool was dialyzed with buffer A and then loaded onto a Q-Sepharose^®^ Fast Flow column (GE Healthcare, Danderyd, Sweden). The recombinant protein, AcCel12B, was eluted using buffer A containing a linear gradient (0–500 mM) of NaCl. The eluted protein was dialyzed and analyzed by 15% SDS-PAGE stained with Coomassie Blue. Protein concentrations were measured using Bradford assay [49] with bovine serum albumin as the standard.

### 3.3. Enzyme Activity Assay

The endoglucanase activity of AcCel12B was determined by incubating the enzyme with CMC (1%, *w*/*v*) and RAC [50] (0.5%, *w*/*v*) at 70 °C for 5 and 15 min, respectively. Sodium acetate buffer (50 mM, pH 4.5) was used for all assay steps unless otherwise described. The concentration of reducing ends was determined by the 3,5-dinitrosalicylic acid (DNS) method using glucose as a standard, as previously described [51]. The absorbance at 540 nm was measured using a UV-2550 spectrophotometer (Shimadzu, Kyoto, Japan). One unit (U) of cellulase activity was defined as the amount of enzyme catalyzing the release of 1 µmol of reducing sugar per min.

### 3.4. Enzyme Characterization of AcCel12B

The optimal temperature for AcCel12B activity was determined at temperatures ranging from 30 to 85 °C and pH 4.5. The effect of pH on activity was carried out by incubating the enzyme with low viscosity CMC (1%, *w*/*v*) or RAC (0.5%, *w*/*v*) in buffers of different pH. Sodium acetate buffer (50 mM, pH 3–6) and sodium phosphate buffer (50 mM, pH 6–7) were used. Thermostability assessment of AcCel12B was performed by incubating the enzyme in 50 mM sodium acetate buffer (pH 4.5) for various periods of time at 60, 65, 70, and 75 °C. Enzyme activity was determined by the method described in Section 3.3. The influences of metal ions and chemical agents on enzyme activity were tested using the above methods at pH 4.5 and 70 °C with metal ions and chemical agents added. The final concentrations of metal ions in the reaction mixture were 1 and 10 mM. For substrate specificities determination, CMC (low viscosity), CMC (medium viscosity), β-d-glucan (barley), RAC, Avicel^®^ PH101 (Fluka, Buchs, Switzerland), Sigmacell cellulose type 50 (Sigma-Aldrich, St. Louis, MO, USA), Pachyman, starch, xylan (birch), and galactomannan were used as substrates and enzyme activity was measured. RAC was prepared from Avicel^®^ PH101 as described by Zhang *et al.* [50]. The other materials were purchased from Sigma-Aldrich (St. Louis, MO, USA) or Fluka (Buchs, Switzerland). The kinetic constants (*K*_m_ and *V*_cat_) of AcCel12B activity toward CMC (low viscosity) were determined using Lineweaver-Burk plots of the reciprocal of reaction velocity (1/V) against the reciprocal of the corresponding substrate concentrations (1/S).

### 3.5. Time Course of Hydrolysis

The reactions were carried out in 2 mL Eppendorf plastic tubes on a heating block at 70 °C upon mixing (800 rpm). 200 µL mixtures (in triplicate) containing AcCel12B (0.18 μM) and RAC (0.5%, *w*/*v*) incubated for 0, 5, 10, 15, 20, 25, 30, 35, 40, 60, and 90 min. At the given time points, the tubes were removed and placed in salt-ice mixture to stop the reaction. The insoluble RAC was separated by centrifugation (2 min, 10,000× *g*). The pellet was washed three times with buffer and separated by centrifugation. All of the above steps were performed while maintaining the temperature below 4 °C. The supernatant was collected and the concentration of reducing ends was determined as described in Section 3.3. The concentration of reducing ends in the pellet was also determined. Samples containing thermally denatured enzyme were used as a blank. The concentration of soluble sugars in the supernatant was analyzed by reversed-phase chromatography using a Waters 600 HPLC system (Waters Corporation, Milford, MA, USA) fitted with a XAmide column (4.6 × 250 mm, 5 µm particle size, Acchrom, Beijing, China). The mobile phase was acetonitrile/water (65/35 (*v*/*v*), 0.22 μm filtered and degassed) and the flow rate was 1 mL·min^−1^.

## 4. Conclusions

In this study, the gene *ABK52392* encoding an EG of GH12 from *A. cellulolyticus* 11B was cloned and expressed in *E. coli*. The recombinant enzyme AcCel12B presented optimal activity at pH 4.5 and 75 °C. The enzyme also exhibited stability at high temperature and under acidic conditions. An unusual property of AcCel12B was that it had similar levels of activity toward soluble substrate (CMC) and insoluble substrate (RAC) of 118.3 and 104.0 U·mg^−1^, respectively. The time course of RAC hydrolysis was investigated and the results indicated that a slowdown in hydrolysis after the early stage of incubation was dependent on the rate of IRE generation.

## Supplementary Materials

Supplementary materials can be found at http://www.mdpi.com/1422-0067/16/10/25080/s1.
