# Supplementary material for: Cloning, Expression, and Characterization of a Thermophilic Endoglucanase, AcCel12B from Acidothermus cellulolyticus 11B
Source: Int J Mol Sci. 2015 Oct 22;16(10):25080–95. doi: 10.3390/ijms161025080 (PMC4632791; doi:10.3390/ijms161025080)
Supplement: Supplementary file 1 [file ijms-16-25080-s001.pdf]

Supplementary Information

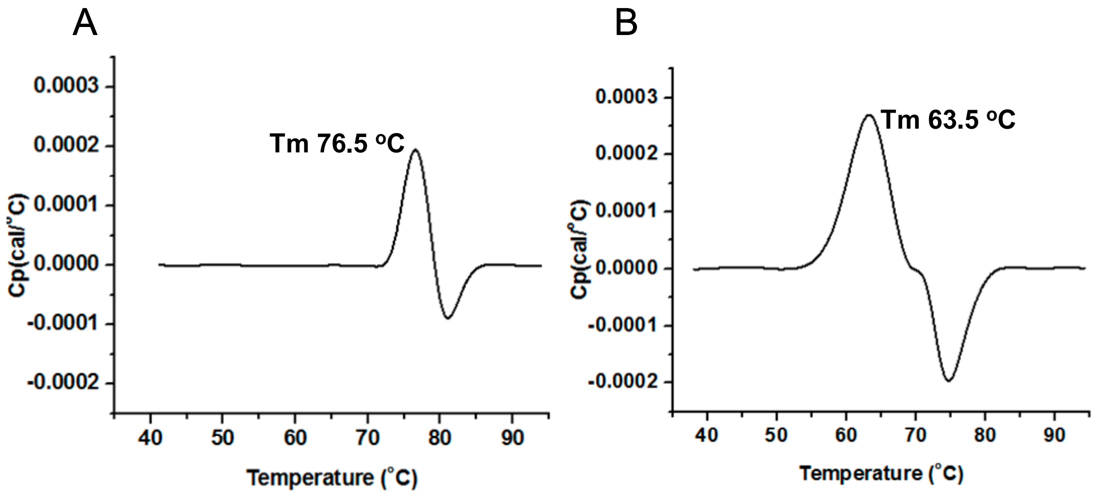

Figure S1. Representative DSC profile of AcCel12B at **A**. 10 mM sodium acetate buffer (pH 4.5) and **B**. 10 mM sodium phosphate buffer (pH 7.0). The protein concentration was 1 mg/mL and excessive heat capacity curves were recorded using MicroCal VP-DSC at a heating rate of 1 °C/min. The experimental data were base-line-corrected and analyzed using the MicroCal ORIGIN software (OriginLab Corporation, USA).

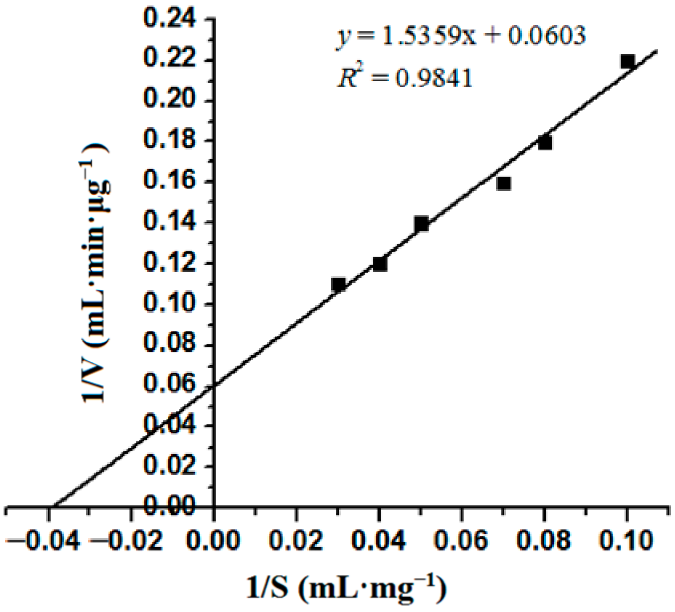

Figure S2. Effect of CMC concentration on the activity of AcCel12B. The activity of AcCel12B was measured in the presence of the indicated concentrations of CMC at 70 °C. The Lineweaver-Burk plot of velocity versus various fixed CMC concentrations was shown and fitted linearity. Each value represents the mean of triplicate measurements and varies from the mean by not more than 10%.

Table S1. Effects of metals ions and chemical agents on the activity of AcCel12B.

**Metal Ion or Chemical Agent**	**Relative Activity (%)**	
Control	100	100
-	1 mM	10 mM
Na^+	101 ± 2.9	101 ± 4.0
K^+	99 ± 1.2	101 ± 3.5
Li^+	100 ± 2.3	99 ± 8.7
NH_4^+	99 ± 1.9	102 ± 3.0
Co^{2+}	112 ± 1.8	108 ± 0.9
Cd^{2+}	93 ± 4.1	84 ± 2.8
Ba^{2+}	99 ± 3.6	102 ± 5.2
Mg^{2+}	101 ± 3.1	99 ± 4.5
Zn^{2+}	98 ± 1.9	98 ± 2.5
Mn^{2+}	97 ± 4.2	104 ± 2.9
Ca^{2+}	100 ± 4.1	102 ± 1.5
EDTA	90 ± 2.7	87 ± 2.6
DTT	102 ± 5.2	99 ± 1.1
-	1% (*v*/*v*)	10% (*v*/*v*)
Tween 20	89 ± 4.7	73 ± 2.6
